# Sevelamer carbonate crystal associated colitis in a hemodialysis patient: a case report

**DOI:** 10.1093/omcr/omag175

**Published:** 2026-09-14

**Authors:** Omair Bseiso, Ma’moun Qawasmeh, Anas Zahdeh, Obay Isayed, Abrar Omari, Motaz Natsheh

**Affiliations:** Faculty of Medicine, Hebron University, Hebron, West Bank, Palestine; Department of Nephrology, Hebron Governmental Hospital (Al-Alia), Hebron, West Bank, Palestine; Faculty of Medicine, Hebron University, Hebron, West Bank, Palestine; Department of Internal Medicine, An-Najah National University, Nablus, West Bank, Palestine; Prince Hamzah Hospital, Amman, Jordan; Department of Pathology, Al Ahli Hospital, Hebron, West Bank, Palestine

**Keywords:** Sevelamer carbonate, crystal colitis, end-stage renal disease, hemodialysis, phosphate binder, medication-induced colitis

## Abstract

Sevelamer carbonate, a non-calcium phosphate binder widely used in end-stage renal disease (ESRD), is an increasingly recognized but underdiagnosed cause of medication-induced colitis through gastrointestinal crystal deposition. We report a 56-year-old Palestinian male with ESRD on hemodialysis who presented with a one-month history of watery non-bloody diarrhea, vomiting, and diffuse abdominal pain. He had been repeatedly misdiagnosed with amebic colitis and treated with metronidazole without improvement. Colonoscopy revealed diffuse patchy erythema throughout the colon with right-sided predominance. Histopathology confirmed sevelamer-induced active colitis, demonstrating characteristic brownish fish scale-like crystals with active neutrophilic infiltrate. Sevelamer carbonate was discontinued with subsequent clinical improvement. This case highlights a clinically important diagnostic pitfall in resource-limited settings where amebic colitis is endemic and empiric metronidazole is commonly the first-line response to chronic diarrhea in dialysis patients.

## Introduction

Hyperphosphatemia is a near-universal complication of ESRD, and its correction is important to reduce cardiovascular mortality, vascular calcification, and secondary hyperparathyroidism. Phosphate binders are a cornerstone of management when dietary restriction and dialysis are insufficient. Sevelamer carbonate is a non-calcium, non-absorbable anion-exchange resin widely prescribed for this purpose [1].

While common GI adverse effects of sevelamer include nausea, vomiting, diarrhea, and constipation, serious mucosal injury from crystal deposition is increasingly reported. Documented severe events include ulceration, colitis, ischemic-type injury, obstruction, necrosis, and perforation [2].

Histologically, sevelamer-associated injury is identified by characteristic nonpolarizable crystals with broad, curved, irregular “fish-scale” morphology and a two-toned yellow-brown or rusty appearance on hematoxylin and eosin (H&E) staining. These findings are clinically crucial because the resulting colitis can closely mimic infectious colitis, ischemic colitis, inflammatory bowel disease, or nonspecific uremia-related GI symptoms, leading to delayed or missed diagnosis [3]. Sevelamer-associated gastrointestinal injury was first described in 2008, and awareness of this entity has since grown steadily [4].

We report a case of sevelamer carbonate-associated colitis in a hemodialysis patient presenting with chronic watery diarrhea, vomiting, and abdominal pain, with the diagnosis substantially delayed by repeated misidentification as infectious gastroenteritis.

## Case report

A 56-year-old Palestinian man with ESRD on regular hemodialysis for approximately one year presented with a one-month history of progressively worsening watery non-bloody diarrhea (approximately 10 episodes/day), vomiting, and diffuse abdominal pain. His medical history included hypertension, ischemic heart disease, and diabetes mellitus. Current medications included amlodipine, doxazosin, carvedilol, methyldopa, and sevelamer carbonate.

Sevelamer carbonate had been initiated after starting hemodialysis for hyperphosphatemia (initial serum phosphorus 9.5 mg/dL) at a dose of 800 mg four times daily. The patient had been receiving sevelamer carbonate for approximately three months before the onset of gastrointestinal symptoms. Serum phosphorus levels fluctuated during treatment. The patient had attended the emergency department on multiple prior occasions with identical complaints and had been empirically treated for presumed amebiasis with metronidazole without any improvement.

Upon admission, the patient received supportive management including intravenous fluids, electrolyte monitoring, antiemetic therapy, and optimization of his hemodialysis schedule. Stool studies were obtained and infectious causes of chronic diarrhea were investigated. Given the persistence of symptoms despite prior antimicrobial treatment and the unrevealing initial workup, a gastroenterology consultation was obtained for further evaluation.

Differential diagnoses included infectious colitis (particularly amebic colitis and *Clostridioides difficile* infection), inflammatory bowel disease, ischemic colitis, medication-induced colitis, and uremia-related gastrointestinal symptoms. Infectious causes became less likely because stool studies and *C. difficile* testing were negative and the patient failed to improve despite repeated courses of metronidazole. Inflammatory bowel disease was considered less likely in the absence of characteristic endoscopic findings, while histopathological examination ultimately demonstrated characteristic sevelamer crystals, confirming medication-induced colitis.

**Figure 1:**
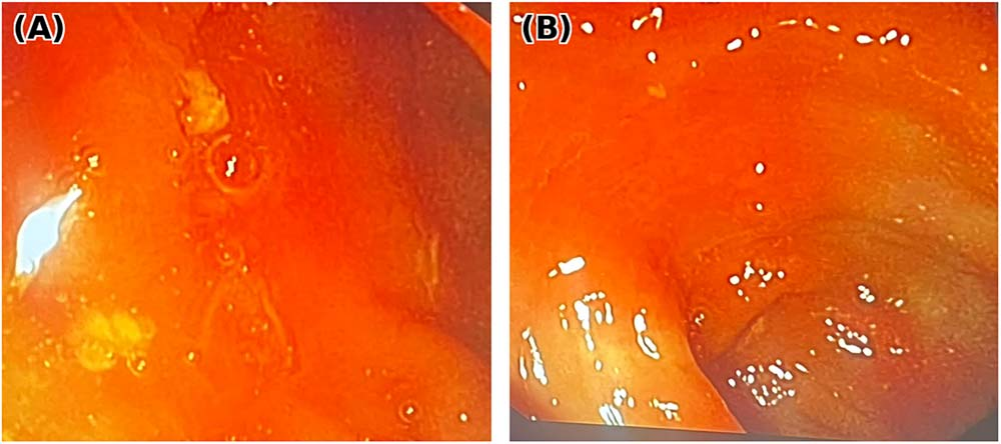
Colonoscopic images demonstrating diffuse patchy erythema of the colonic mucosa. (A) and (B) show erythematous, oedematous mucosa with loss of the normal vascular pattern and patchy erythema, most pronounced in the right colon. No discrete ulcers, pseudomembranes, or masses were identified.

Given persistent symptoms despite antimicrobial therapy and a negative infectious evaluation, colonoscopy was performed on day 1 using a standard high-definition colonoscope under propofol sedation with good bowel preparation. The rectum showed internal haemorrhoids with otherwise normal mucosa. The sigmoid colon, descending colon, splenic flexure, transverse colon, hepatic flexure, ascending colon, and caecum all demonstrated patchy erythema, most pronounced in the right colon (Figure 1). The terminal ileum was normal. Random biopsies were obtained from multiple colonic segments.

**Table 1:** Summary of the patient’s clinical course.

**Time / Period**	**Clinical event**
~1 year before presentation	Patient initiated regular hemodialysis for ESRD.
After hemodialysis initiation	Sevelamer carbonate 800 mg four times daily started for hyperphosphatemia (initial serum phosphorus 9.5 mg/dL). The patient had been receiving sevelamer for approximately three months before the onset of GI symptoms. Phosphorus levels fluctuated during treatment.
~1 month before admission	Onset of intermittent watery non-bloody diarrhea, vomiting, and diffuse abdominal pain, progressively worsening to ~10 episodes/day.
Prior to current presentation	Multiple emergency department visits. Empirically treated for presumed amebiasis with metronidazole; no clinical improvement.
Hospital Day 1	Colonoscopy: patchy erythema throughout entire colon (right-sided predominance), internal haemorrhoids in rectum, normal terminal ileum. Random biopsies obtained. Sevelamer carbonate discontinued and switched to calcium carbonate.
Hospital Day 3	Colonic biopsy specimens received at pathology laboratory.
Hospital Day 4	First noticeable clinical improvement: reduction in diarrheal frequency following sevelamer discontinuation.
Hospital Day 5	Pathology report: sevelamer-induced active colitis. Preserved crypt architecture; neutrophilic infiltrate with cryptitis and crypt abscesses; brownish fish scale-like crystals consistent with sevelamer. Negative for malignancy, granulomas, parasites, viral changes.
Hospital Day 7 (discharge)	Marked reduction in diarrhea and resolution of abdominal pain. Discharged in stable condition. Outpatient nephrology and gastroenterology follow-up arranged.

**Figure 2:**
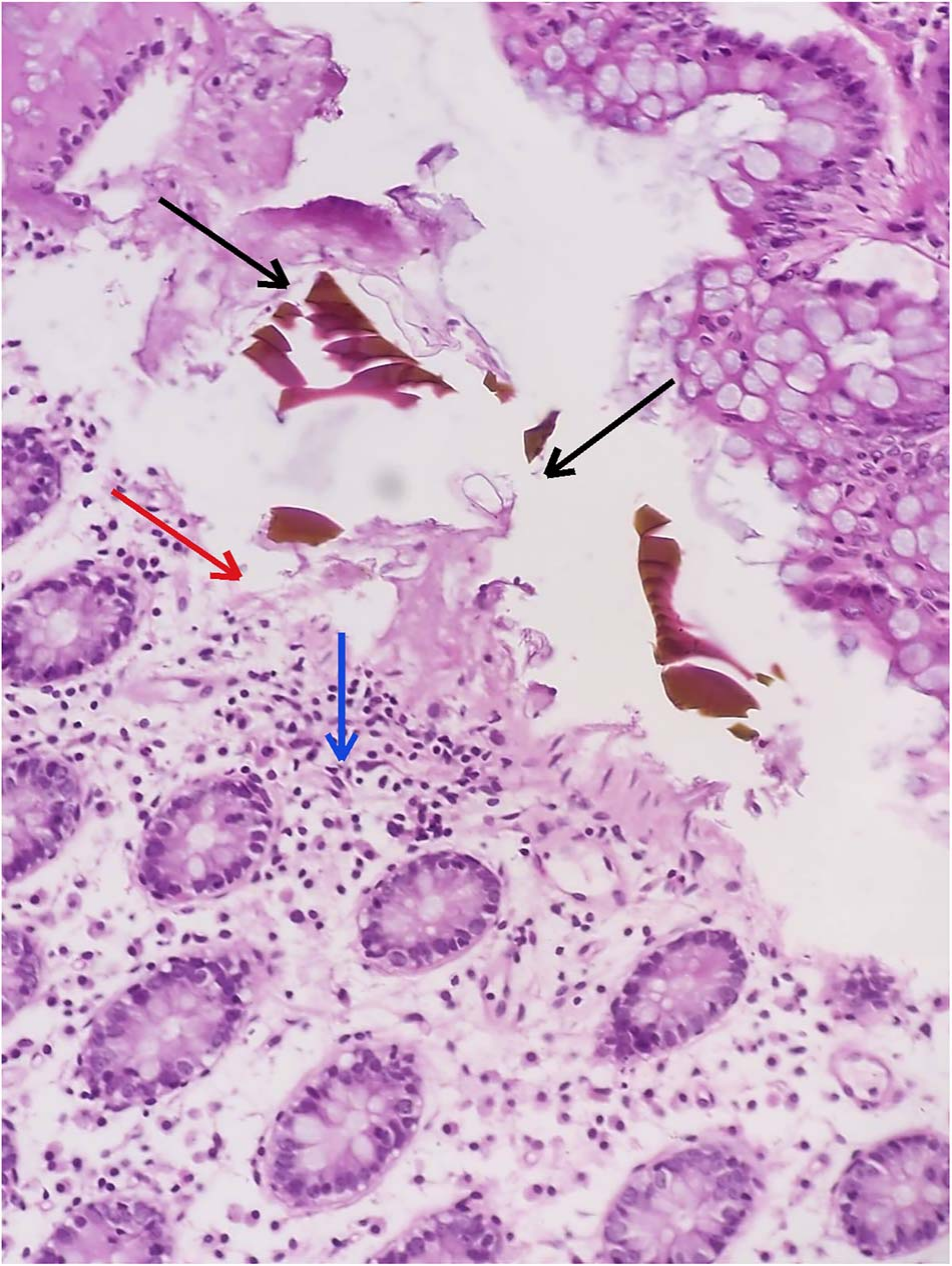
Colonic biopsy, haematoxylin and eosin stain. Preserved colonic crypt architecture with goblet cells is evident. Black arrows indicate characteristic brownish fish scale-like sevelamer crystals. The red arrow indicates neutrophilic inflammatory infiltrate within the lamina propria. The blue arrow indicates cryptitis. These findings are consistent with sevelamer-associated active colitis.

Colonic biopsy specimens were received at the pathology laboratory on hospital day 3 and reported on day 5. Two fragments of grayish tissue measuring 0.9×0.8 cm in aggregate were totally submitted in one cassette. Histopathological examination on H&E-stained sections (Figure 2) demonstrated preserved colonic crypt architecture; increased neutrophilic infiltrate in the lamina propria with cryptitis and crypt abscesses consistent with active colitis; superficial ulcers containing brownish, angular, fish scale-like crystalline material morphologically consistent with sevelamer crystals; and negative for viral cytopathic changes, parasites, granulomas, dysplasia, or malignancy. The pathological diagnosis was ***sevelamer-induced active colitis.***

**Table 2:** Laboratory findings, vital signs, and clinical parameters during hospitalization.

**Parameter**	**Admission (Day 1)**	**After discontinuation (Day 4)**	**Discharge (Day 7)**	**Reference range**
Blood pressure (mmHg)	112/70	Stable	Stable	90-120 / 60-80
Heart rate (beats/min)	96	Improved	Normal	60-100
Respiratory rate (/min)	18	18	18	12-20
Temperature (°C)	36.9	Afebrile	Afebrile	36.5-37.5
Oxygen saturation (%)	99	99	99	≥95
Hemoglobin (g/dL)	12.5	-	-	13.5-17.5
WBC (×10^9^/L)	4.1	-	-	4.0-11.0
Platelets (×10^9^/L)	78	-	-	150-400
Sodium (mmol/L)	135	-	-	135-145
Potassium (mmol/L)	5.8	Improved	Stable	3.5-5.1
Serum albumin (g/dL)	Not measured	-	-	3.5-5.0
Arterial blood gas (pH)	7.48^a^	Not repeated	-	7.35-7.45
Stool analysis	Negative	-	-	Negative
Stool osmolality	Not performed	-	-	-
Fecal calprotectin	Not performed	-	-	<50 μg/g
Fecal elastase	Not performed	-	-	>200 μg/g
Diarrhea frequency	~10 episodes/day	Markedly reduced	Resolved	-

^a^Arterial blood gas was performed during a previous emergency department visit and demonstrated mild respiratory alkalosis attributed to dehydration; it was not repeated during the current admission as it was not clinically indicated.

Based on the combination of chronic sevelamer carbonate exposure, persistent unexplained GI symptoms unresponsive to metronidazole, colonoscopic findings of diffuse patchy colonic erythema with right-sided predominance, and histopathologic identification of characteristic sevelamer crystals in injured colonic mucosa with active neutrophilic colitis, a diagnosis of sevelamer carbonate-associated crystal colitis was established.

Sevelamer carbonate was promptly discontinued following the diagnosis. The patient received supportive care including intravenous fluid resuscitation, electrolyte correction (given hyperkalaemia at 5.8 mmol/L in the context of ESRD), antiemetics, and optimisation of his haemodialysis schedule. For ongoing phosphate management, he was transitioned to calcium carbonate with reinforcement of dietary phosphate restriction. Following sevelamer withdrawal, clinical improvement became noticeable within 4 days of discontinuation, with progressive reduction in diarrheal frequency thereafter. Abdominal pain also improved significantly during the first week. The patient was discharged on hospital day 7 in stable condition with scheduled outpatient follow-up. At follow-up, he remained symptom-free with no further emergency department visits for GI complaints.

A repeat colonoscopy with biopsy was not performed because the patient experienced complete clinical resolution following withdrawal of sevelamer carbonate; consequently, macroscopic and histological mucosal healing were not formally documented. Follow-up was based on clinical assessment and symptom monitoring. The patient was counseled at discharge to seek gastroenterology review should symptoms recur, and surveillance colonoscopy was deferred in the absence of ongoing symptoms or alarm features.

## Discussion

Sevelamer carbonate is widely prescribed for hyperphosphatemia in ESRD patients because it provides effective phosphate binding without the calcium loading and cardiovascular risk associated with calcium-based binders. However, it is increasingly recognized as a cause of serious GI mucosal injury through crystal deposition, an entity that may be significantly under-reported due to its overlap with more common GI diagnoses [1, 2].

The present case illustrates the substantial diagnostic challenge posed by sevelamer-associated colitis. The patient experienced more than one month of misdiagnosis, with repeated emergency department visits, empiric metronidazole therapy, and no clinical improvement. This pattern is consistent with previously reported cases in which sevelamer colitis was initially attributed to infectious gastroenteritis, ischemic colitis, or inflammatory bowel disease [5, 6].

The endoscopic appearance in our patient, with diffuse patchy erythema throughout the entire colon with right-sided predominance and a normal terminal ileum and rectum, is consistent with, though not specific to, sevelamer-associated colitis. The right-sided predominance has been noted in prior reports and may reflect higher local concentrations of sevelamer crystals in the proximal colon, greater mucosal surface area, longer transit time, or regional differences in mucosal blood supply and susceptibility [5, 6]. In contrast, Tieu et al. reported sevelamer-associated ulcers confined to the rectosigmoid region, highlighting that crystal deposition and mucosal injury can occur at any colonic segment, with no single anatomical distribution being pathognomonic [7].

Histopathology is the gold standard for diagnosis. The characteristic finding of nonpolarizable, brownish, angular, “fish scale”-like crystals with a two-toned yellow-brown or rusty appearance on H&E staining, often embedded within superficial ulcers or necrotic mucosa, is highly specific when identified in the appropriate clinical context [3]. In our case, these crystals were found in association with active neutrophilic colitis, cryptitis, and crypt abscesses, with an overall preserved crypt architecture and no features of dysplasia, malignancy, or granulomatous inflammation. Importantly, the pathologist should be informed of sevelamer exposure, as the crystals may be overlooked or misattributed to other foreign material if not specifically sought.

The clinical spectrum of sevelamer-associated GI injury is wide. Published cases range from mild diarrhea and abdominal pain to overt GI bleeding, pseudotumor formation, colonic perforation, and life-threatening hemorrhage [8–10]. Our patient’s presentation was notably dominated by chronic watery non-bloody diarrhea without overt bleeding or acute abdomen, emphasizing that clinically significant sevelamer injury need not declare itself dramatically.

The pathogenesis of sevelamer-induced mucosal injury is incompletely understood. Proposed mechanisms include direct mechanical irritation by insoluble crystalline resin particles, local ischemia from crystal-induced vascular compression, impaired mucosal healing in the uremic environment, and increased vulnerability of the dialysis patient’s GI mucosa due to comorbid vascular disease, altered motility, and polypharmacy [8, 10]. In this patient, diabetes mellitus and ischemic heart disease may have further compromised mucosal perfusion and healing capacity, potentiating sevelamer-associated injury.

Of particular relevance to clinical practice in low- and middle-income countries and resource-limited settings is the pattern of misdiagnosis observed in this case. In the Palestinian West Bank and similar regions, amebic colitis remains a common clinical concern, and empiric metronidazole therapy is frequently initiated for patients presenting with chronic watery diarrhea. Sevelamer-associated colitis has not, to our knowledge, been previously reported from this geographic context. The clinical overlap between sevelamer colitis and amebic colitis, both presenting with chronic diarrhea, diffuse colonic mucosal changes, and absence of systemic toxicity, creates a specific and underappreciated diagnostic trap in ESRD patients from these regions. Crucially, the absence of response to metronidazole, rather than being recognized as a diagnostic clue, may lead to repeated treatment courses and further delay. This case therefore draws attention to the need for gastroenterologists and nephrologists practicing in similar settings to include medication-induced colitis, specifically sevelamer crystal colitis, in the differential diagnosis of chronic diarrhea in dialysis patients, before attributing symptoms to endemic infectious causes.

Management is centered on early recognition and prompt discontinuation of sevelamer. In most published cases, drug withdrawal combined with supportive care results in clinical improvement. Phosphate management must continue after sevelamer withdrawal, and alternative strategies such as calcium carbonate, lanthanum carbonate, sucroferric oxyhydroxide, or ferric citrate should be individualized to the patient’s overall profile [8, 10].

A limitation of this report is the inherent restriction of single-case causality inference. Additionally, confirmatory crystal staining with periodic acid-Schiff with diastase was not performed. Fecal calprotectin, fecal elastase testing, and arterial blood gas analysis were not performed during the current admission as they were not considered clinically necessary once the histopathological diagnosis was established; notably, the patient exhibited no weight loss or steatorrhea to suggest malabsorption or pancreatic exocrine insufficiency. Nevertheless, the temporal association with sevelamer exposure, the thorough exclusion of infectious and inflammatory etiologies, the characteristic histopathologic findings on H&E staining, and the clinical improvement following drug withdrawal collectively constitute strong evidence of sevelamer carbonate-associated crystal colitis.

Greater awareness of this entity among clinicians and pathologists is essential to reduce diagnostic delay, avoid unnecessary antimicrobial therapy, and prevent progression to severe complications. To our knowledge, this represents the first reported case of sevelamer carbonate-associated crystal colitis from Palestine and one of the very few cases reported from the Arab world or resource-limited settings where amebic colitis is endemic. Raising awareness of this entity in these clinical contexts may prevent unnecessary antimicrobial exposure, repeated emergency visits, and progression to severe GI complications.
